# Does applied ultrasound prior to laparoscopy predict the existence of intra-abdominal adhesions?

**DOI:** 10.3906/sag-1910-61

**Published:** 2020-04-09

**Authors:** Hilal USLU YUVACI, Arif Serhan CEVRİOĞLU, Yasemin GÜNDÜZ, Nermin AKDEMİR, Alper KARACAN, Ünal ERKORKMAZ, Abdurrahim KESKİN

**Affiliations:** 1 Department of Obstetrics and Gynecology, Faculty of Medicine, Sakarya University, Sakarya Turkey; 2 Department of Radiology, Faculty of Medicine, Sakarya University, Sakarya Turkey; 3 Department of Biostatistics, Faculty of Medicine, Sakarya University, Sakarya Turkey

**Keywords:** Adhesion, laparoscopy, ultrasonography, visceral sliding sign

## Abstract

**Background/aim:**

The purpose of this study was to evaluate the efficacy of trans-abdominal ultrasonography (USG), a noninvasive diagnostic tool, in predicting the presence of intraabdominal adhesions, especially near the trocar entry area, to provide safe surgical access to the abdomen.

**Materials and methods:**

Fifty-nine women with a previous history of open abdominal surgery (group A) and a group of 91 women with no previous history of surgery (group B) underwent dynamic ultrasound evaluation of the abdominal fields before laparoscopic operations. The anterior abdominal wall was divided into six quadrants: right upper, right lower, left upper, left lower, suprapubic, and umbilical. Adhesions were evaluated by surgeons during the operation and by radiologists using USG prior to the operation. Visceral organ movements greater than 1 cm was defined as normal visceral slide (positive test), with less than 1 cm of movement defined as abnormal visceral slide (negative test). Sliding test measures movements of omental echogenicity or a stable echogenic focus that corresponds to intestine peritoneal echogenicity that underlies abdominal wall during exaggerated inspiration and expiration. Adhesions observed during surgery were evaluated on a four-point scale, with 0 indicating no adhesions present, 1 indicating the presence of a thin, filmy avascular adhesion, 2 indicating the presence of a dense and vascular adhesion, and 3 indicating adhesions that connect surrounding organs with the overlying peritoneal surfaces. The McNemar test was used to compare the results of USG and laparoscopy for each measure.

**Results:**

We found that preoperative USG was successful in identifying adhesions [sensitivity, 96.39% (95% CI 89.8–99.2); specificity, 97.43%]

**Conclusion:**

Preoperative ultrasound examination of the abdominal wall may enhance the safety of abdominal entry during laparoscopic operations.

## 1. Introduction

Intraabdominal (IA) organs move freely against the abdominal wall during respiration. This phenomenon is known as visceral sliding. Previous abdominal surgery and peritonitis often cause IA adhesions, which prevents or reduces visceral sliding [1]. Sigel et al. reported that a reduction in visceral sliding shown by transabdominal USG can be a reliable marker of abdominal adhesions [2]. The absence of visceral sliding is associated with adhesion of organs to the abdominal wall [3].

Previous studies have shown that adherence between the abdominal wall and visceral organs occurs in 25–50% of patients undergoing surgery [4–6]. IA adhesions may be of the thin, filmy, or dense type [7].

IA adhesions may cause chronic abdominal pain, infertility, intestinal obstruction, and dyspareunia [8,9]. It has been reported that approximately one-third of all patients with a history of abdominal surgery have been referred for complications related to IA adhesions during the 10-year postoperative period [10]. Furthermore, adhesions between the anterior abdominal wall and visceral organs may also lead to injury to these organs at the beginning of operations [11–13]. In particular, organ injury that occurs during trocar insertion is one of the most preventable potentially serious complications [14]. Thus, surgeons are in dire necessity of a noninvasive and reliable test that could improve the preoperative diagnosis of adhesions between abdominal wall and organs. Marine et al. [15] demonstrated that IA adhesions can be recognized ultrasonographically just prior to trocar entry after pneumoperitoneum.

The goal of our study was to evaluate the predictive efficacy of preoperative TA ultrasound for the detection of IA adhesions, especially under the trocar entry areas. 

## 2. Materials and methods

### 2.1. Patients and settings

This study was approved by the Clinical Research Ethics Committee of our university (Project ID number: 17/01/2014, 54).

**Figure 1:**
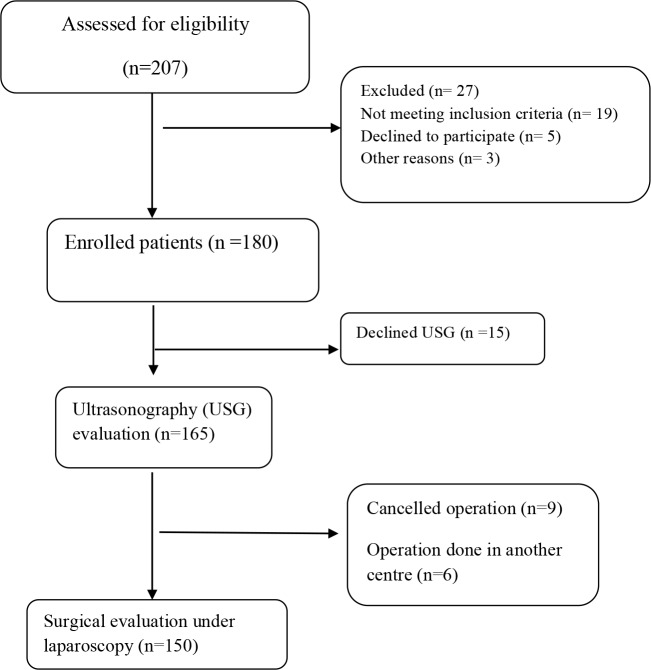
Participant flow diagram for ultrasound visceral slide.

Recruitment of patients for this prospective laparoscopic surgery study took place at the Department of Obstetrics and Gynecology between December 2016 and December 2017. A total of 208 women were examined and scheduled for the study, and 150 women’s data could be evaluated at the end of the study (Figure 1).

Inclusion criteria were an age of 18 years or older, a history of prior laparoscopic or open intraabdominal surgery, a planned laparoscopy (LS) with any gynecologic indication, and the ability to understand the study and provide informed consent. The sole exclusion criterion was a history of abdominal surgery within the past 6–8 weeks. The LS indications were uterine fibroids, infertility, adnexial mass, endometrioma, chronic pelvic pain, adenomyosis, tubal ligation request, and ectopic pregnancy out of emergency.

We obtained basic demographic information including age, body mass index (calculated as weight (kg)/[height (m)]2), gravidity, parity, medical history, and surgical history, obtained through interview and by reference to the medical records of the participants.

### 2.2. Method

Prior to elective surgery, USG was performed to evaluate visceral slide. Evaluation was done by trained, staff radiologists (YG, AK) at the time of the patient’s preoperative visit. USG was performed using an ultrasonography device (Aplio 400; Toshiba, Japan), with a 5.2-MHz abdominal superficial transducer placed in the axial plane at the abdomen. Patients were examined in the supine position and ultrasound gel was used to achieve acoustic coupling between the transducer and the skin. 

The ultrasound scan focused on the anterior abdominal wall, which was evaluated for the presence of visceral slide in six predefined segments: right upper quadrant (RUQ), right lower quadrant (RLQ), left upper quadrant (LUQ), left lower quadrant (LLQ), suprapubic quadrant (SPQ), and umbilical quadrant (UQ). The umbilical zone was defined as the area within a 5-cm radius of the umbilicus. 

The patients were asked to take normal and exaggerated breaths for the evaluation of spontaneous and induced visceral slide, respectively. In cases of uncooperative patients, organ movement was induced by manual abdominal ballottement (induced visceral slide). During USG, attention was paid to a distinct hyperechogenic area just beneath the anterior abdominal wall peritoneum. The maximum excursion of this area was measured and recorded in the six predefined segments.

**Figure 2:**
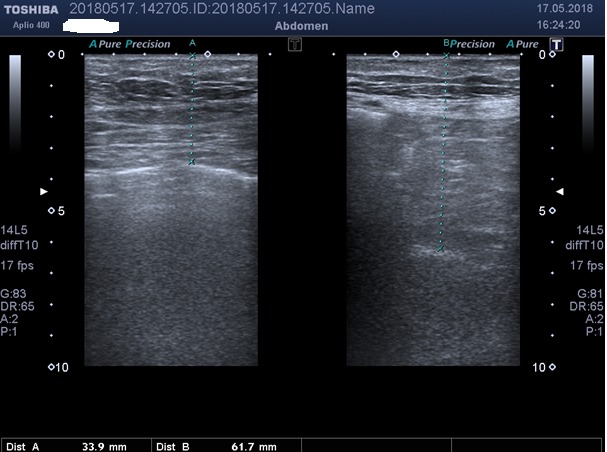
The longitudinal distance the viscera travelled as visualized on ultrasonographyfrompoint A topoint B (slidinng sign +).

A stable echogenic focus corresponding to the omentum or intestine was identified, and the distance travelled by the focus was recorded while the participant performed an exaggerated inspiration and expiration. The visceral slide, i.e. the longitudinal distance that the viscera travelled from point A to point B, as visualized on USG, was noted (Figure 2). Movement of the viscera greater than 1 cm was defined as normal visceral slide (positive test), and movement of less than 1 cm was defined as abnormal visceral slide (negative test), as previously established by Tu et al. [3].

Operative findings during surgery were recorded by the operating surgeon. Surgeons ASC, NA, and HUY performed the laparoscopic operations and assessed the six predefined segments for intraabdominal adhesions in all 150 patients during the surgery. The following scoring system [7] was used to classify the adhesion severity: Score 0, no adhesions present; Score 1, presence of thin, filmy avascular adhesions; Score 2 presence of dense and vascular adhesions; and Score 3, at least one adhesion connecting the surrounding organs to the peritoneal surfaces.

### 2.3. Statistics

The primary end-point was the diagnostic accuracy of USG. Effect size was calculated on the assumption that the ultrasound diagnostic consistency ratio was 0.81, based on the existing literature [1]. The required sample size was calculated as 126, assuming an alpha value of 0.05 and power of 0.80. We enrolled 200 subjects to cover potential loss of patients to follow-up. Power analysis was performed using PASS software (ver. 13.0; NCSS, LLC., Kaysville, UT, USA).

Descriptive analyses were performed to yield information on the general characteristics of the study population. The sensitivity, specificity, negative predictive value (NPV), and positive predictive value (PPV) (with 95% confidence interval [CI]) for USG were calculated for predicting adhesions, using laparoscopy (LS) as the gold standard. The McNemar test was used to compare the results of USG and LS for each measure. Categorical data are presented as counts (n) and percentages (%). Continuous data are presented as means ± standard deviation. A P-value <0.05 was considered to be significant. Analyses were performed using IBM SPSS Statistics commercial software (ver. 23.0; IBM Corp., Armonk, NY, USA).

## 3. Results

The demographic information and preoperative characteristics of the participants are presented in Table 1. Fifty-nine women (39.3%) who had previously undergone abdominal surgery received LS for benign gynecological indications. Scars from previous operations included Pfannenstiel scars (50.8%), subumblical median incisions (3.4%), subumblical or upperumblical median incisions (1.7%), McBurney incisions (15.3%), and laparoscopic hole scars (28.8%). Ninety-one women (60.7%) who did not have prior history of abdominal operation and were also operated with LS for benign gynecological indications comprised the control group.

**Table 1:** Patient demographics and preoperative characteristics of 150 patients assessed with the visceral slide technique.

		Study group(n = 59, 39.3%)	Control group(n = 91, 60.7%)	Age (years)	
39.27 ± 8.86	38.53 ± 11.61	Gravida		2.03 ± 1.65
2.16 ± 2.0	Parity		1.73 ± 1.42	1.74 ± 1.61
BMI*		33.29 ± 4.15	26.06 ± 4.12	Operation duration (minutes)	
92.54 ± 29.94	86.93 ± 32.01	Scar type	Phannenstiel
30 (50.8)			SUM	2 (3.4)
	SUM-UUM		1 (1.7)	
Mc Burney	9 (15.3)			Laparoscopic trocar hole
17 (28.8)		

BMI, body mass index, kg/m^2^; SUM, subumblical median incision; SUM-UUM, sub- and upper umblical median incision

Indications for surgery included uterine fibroids, infertility, adnexal mass, endometrioma, chronic pelvic pain, adenomyosis, tubal ligation request, and ectopic pregnancy out of emergency. The LS operations performed were myomectomy (27.3%), hysterectomy (3.3%), hysterectomy and bilateral salpingo-oophorectomy (22.7%), cystectomy (26.7%), salpingectomy (4.7%), diagnostic LS (14%), and tubal ligation (1.3%).

Findings at the time of operation are shown in Table 2. Adhesions seen during the operation were scored as 0 for no adhesion, 1 for thin, filmy avascular adhesions, 2 for dense and vascular adhesions, and 3 for adhesions connecting surrounding organs with the peritoneal surfaces. All findings were recorded by the operating surgeon. 

**Table 2:** Intraoperative findings.

Adhesions during surgery	Score 0	99 (66%)
	Score 1	30 (20%)
	Score 2	20 (13.3%)
	Score 3	1 (0.7%)

Score 0, no adhesion; Score 1, thin-film avascular adhesion; Score 2, dense and vascular adhesion; Score 3, adhesion that connects surrounding organs with the peritoneal surfaces

In Tables 3–5, intraoperative findings were evaluated separately in six predefined regions. In the study group, the visceral sliding test performed using USG had a sensitivity of 96.39% (95% CI 89.8–99.2), specificity of 97.43% (95% CI 96.1–98.4), PPV of 97.43% (95% CI 70.0–86.6), and NPV of 99.6% (95% CI 98.6–99.9) to predict adhesions during the preoperative period (Table 3).

**Table 3:** Diagnostic performance of USG for determining visceral adhesions compared to the gold standard (laparoscopy).

		Visceral adhesion (Laparoscopy)	
		Absent	Present
Visceral adhesion (USG)	Absent	796	3
	Present	21	80
Sensitivity		80/83	96.39, 95% CI (89.8–99.2)
Specificity		796/817	97.43, 95% CI (96.1–98.4)
PPV		80/101	79.2, 95% CI (70.0–86.6)
NPV		796/799	99.60, 95% CI (98.6–99.9)

USG, ultrasonography; PPV, positive predictive value; NPV, negative predictive value; CI, confidence interval

The diagnostic performance of USG, according to intraoperative visceral adhesion type, is described in Table 4. The performance of USG according to the location of adhesions is described in Table 5.

**Table 4:** Diagnostic performance of USG according to intraoperative visceral adhesion type findings.

Adhesion type (LS)		Visceral adhesion (USG)		P
		No (n/%)	Yes (n/%)	
Thin and filmy	No	782 (90.8)	79 (9.2)	<0.001
	Yes	17 (43.6)	22 (56.4)	
Dense	No	784 (91.2)	76 (8.8)	<0.001
	Yes	15 (37.5)	25 (62.5)	
Binding	No	799 (88.9)	100 (11.1)	<0.001
	Yes	0 (0)	1 (100)	

LS, Laparoscopy; USG, ultrasonography; BMI, body mass index

**Table 5:** Diagnostic performance of USG according to adhesion location.

	Adhesion score (LS)		Visceral adhesion (USG)		P
			Absent	Present	
UQ	Score1	Absent	137 (95.1)	7 (4.9)	0.180
		Present	2 (33.3)	4 (66.7)	
	Score 2	Absent	137 (93.2)	10 (6.8)	0.039
		Present	2 (66.7)	1 (33.3)	
	Score 3	Absent	139 (92.7)	11 (7.3)	0.001
		Present	0 (0)	0 (0)	
RLQ	Score 1	Absent	141 (99.3)	1 (0.7)	0.070
		Present	7 (87.5)	1 (12.5)	
	Score 2	Absent	145 (99.3)	1 (0.7)	0.625
		Present	3 (75)	1 (25)	
	Score 3	Absent	148 (98.7)	2 (1.3)	0.500
		Present	0 (0)	0 (0)	
RUQ	Score 1	Absent	123 (83.7)	24 (16.3)	<0.001
		Present	0 (0)	3 (100)	
	Score	Absent	120 (83.3)	24 (16.7)	<0.001
		Present	3 (50)	3 (50)	
	Score3	Absent	123 (82)	27 (18)	<0.001
		Present	0 (0)	0 (0)	
LLQ	Score1	Absent
		143 (96.6)	5 (3.4)	0.453
	Present	2 (100)	0 (0)	
	Score2	Absent	140 (98.6)	2 (1.4)	0.453
		Present	5 (62.5)	3 (37.5)	
	Score3	Absent	145 (96.7)	5 (3.3)	0.063
Present		0 (0)	0 (0)		
LUQ	Score1	Absent	126 (85.1)	22 (14.9)	<0.001
		Present	1 (50)	1 (50)	
	Score2	Absent	127 (88.8)	16 (11.2)	<0.001
		Present	0 (0)	7 (100)	
	Score3	Absent	127 (84.7)	23 (15.3)	<0.001
		Present	0 (0)	0 (0)	
SPQ	Score1	Absent	112 (84.8)	20 (15.2)	0.004
		Present	5 (27.8)	13 (72.2)	
	Score2	Absent	115 (83.3)	23 (16.7)	<0.001
		Present	2 (16.7)	10 (83.3)	
	Score3	Absent	117 (78.5)	32 (21.5)	<0.001
		Present	137 (95.1)	7 (4.9)	

UQ, umbilical quadrant; RLQ, right lower quadrant; RUQ, right upper quadrant; LLQ, left lower quadrant; LUQ, left upper quadrant; SPQ, suprapubic quadrant; Score 0, no adhesion; Score 1, thin-film avascular adhesion; Score 2, dense and vascular adhesion; Score 3, adhesion that connects surrounding organs with the peritoneal surfaces

## 4. Discussion 

The most frightful complications in endoscopic surgery, which is increasingly used nowadays, are those encountered during the first entrance to the abdomen. Safe entry of the abdomen is important, especially in patients who have had previous operations. In these patients, complications due to adhesions at the entrance of the abdomen should be predictable and preventable using noninvasive methods such as USG, which would increase the preference for endoscopic surgical methods even in this risky patient group. 

Preoperative detection of abdominal adhesions remains a difficult task. Nevertheless, there are ongoing studies to identify the optimal noninvasive method of identifying anterior abdominal wall adhesions to provide safer surgical access to the abdomen. Marin et al. performed a study in 39 patients with large abdominal scars, using USG after pneumoperitoneum to guide trocar entry through an abdominal wall region with no adhesions during LS. Their report concluded that USG can be a useful tool for this task [15]. Furthermore, the method of using USG before pneumoperitoneum, developed by Sigel and Kodama et al., is still widely used today [2,16].

In recent years, USG and/or magnetic resonance imaging (MRI) have been proposed as suitable noninvasive tools for the evaluation of abdominal wall adhesions [2,17–20]. Although advanced imaging techniques such as MRI and multislice computerized tomography (CT) have been shown to be valuable for noninvasive diagnosis of abdominal anterior wall adhesions, these methods are both time-consuming and expensive [18,19].

In our study, similar to Sigel et al. [2], we divided the abdomen into six quadrants, and the adhesions were evaluated with USG before and during the operation. We found that preoperative USG was successful in identifying adhesions [sensitivity, 96.39% (95% CI 89.8–99.2); specificity, 97.43%] (Table 3). Meanwhile, Kolecki et al. reported sensitivity and specificity values of USG of 90% and 92%, respectively, in visceral slide evaluations to predict adhesions [21].

We scored intraoperative adhesions identified during LS (Table 2). All of the intraabdominal adhesions screened by preoperative USG were predicted with significant accuracy (Table 4). This may be because better images were achieved thanks to technological advances in the field of radiology. Furthermore, we believe that individual patient effects may have affected our results, including the provision of proper instructions, sufficient respiration, and a reasonable level of cooperation during USG. All USG examinations were made by skilled radiologists who perform an average of 50 or more USG examinations daily.

Nezhat et al. found that evaluations of visceral sliding using preoperative USG in the office setting versus periumbilical ultrasound-guided saline infusion immediately after anesthesia were equivocal. They further concluded that the office visceral test was an easy-to-use test to identify periumbilical adhesions [20]. Unlike our study, USG was performed just before surgery by the surgeons who carried out the operation. 

In the literature, it has been suggested that adhesions in the lower abdominal region may be less accurately assessed by the preoperative visceral sliding technique, as the repulsive force of respiration may be lacking in the lower one-third of the abdomen [3,21].We found that USG recognized adhesions with significantly higher accuracy in the RUQ, LUQ, and SPQ regions compared to the lower regions of the abdomen, i.e. RLQ and LLQ (P < 0.001).

We believe that this study may contribute to the literature by further demonstrating the importance of TAUSG for safe and secure abdominal surgery, especially in light of the increasing use of laparoscopic surgery. Due to the technological advances in ultrasound imaging techniques, detection of even thin, filmy adhesions has become possible in experienced hands. USG is also a more cost-effective measure compared to other imaging modalities, such as MRI or CT. 
